# Rebuilding the critically endangered Yangtze finless porpoise population: successful release from an *ex situ* conservation programme

**DOI:** 10.1098/rsbl.2024.0719

**Published:** 2025-06-04

**Authors:** Jiansong Qiu, Yang Zheng, Fei Fan, Jinsong Zheng, Qiang Zeng, Zijia Xu, Qiang Gao, Kexiong Wang, Songhai Li, Ding Wang, Zhigang Mei

**Affiliations:** ^1^ State Key Laboratory of Lake and Watershed Science for Water Security, Institute of Hydrobiology, Chinese Academy of Sciences, Wuhan, People’s Republic of China; ^2^ University of Chinese Academy of Sciences, Beijing, People’s Republic of China; ^3^ Dalian Ocean University, Dalian, People’s Republic of China; ^4^ Administrative Bureau of Hubei Changjiang Xinluo Baiji National Nature Reserve, Honghu, People’s Republic of China; ^5^ Institute of Deep-sea Science and Engineering, Chinese Academy of Sciences, Sanya, People’s Republic of China

**Keywords:** *Neophocaena asiaeorientalis asiaeorientalis*, *ex situ* conservation, translocation, post-release monitoring, endangered species, biodiversity recovery

## Abstract

*Ex situ* conservation and population reinforcements or reintroductions are vital strategies for protecting endangered species, yet efforts for cetaceans have been notably limited. Through post-release monitoring based on wearable radio tag and passive acoustic methods, we report the first successful release of a critically endangered small toothed whale, the Yangtze finless porpoise (*Neophocaena asiaeorientalis asiaeorientalis*), into the wild. Our monitoring results indicate that, by the third day after release, the two porpoises from the *ex situ* population had joined a local individual and gradually integrated into the core distribution area of the local population. Continuous monitoring by the Yangtze Cetacean Protection Network revealed no recorded deaths among the released porpoises. This successful release demonstrates the potential of this approach as a supplementary measure for the restoration of the Yangtze finless porpoise wild population.

## 1. Introduction

*Ex situ* conservation and following translocation are essential strategies for preventing species extinction and supporting the recovery of wild populations, particularly in response to habitat loss and biodiversity decline due to human activities and global environmental changes [1–3]. Large animals face heightened extinction risks, driven by a combination of environmental pressures and species-specific vulnerabilities [4]. Among these, cetaceans, especially freshwater and nearshore species, are facing an increasing risk of extinction [5]. Current cetacean conservation efforts primarily focus on the establishment of natural reserves, i.e. *in situ* conservation [6–8]. However, for species with small population sizes and at risk of rapid decline, there are insufficient cases to support the role of *ex situ* conservation and subsequent release of *ex situ* individuals into the wild to supplement or re-establish wild populations.

Post-release monitoring (PRM) for cetaceans released into the wild after spending time in captivity or semi-captivity is rare, typically involving individuals from stranding or rescue incidents [9–11], with minimal focus on long-term captive individuals [12,13]. For endangered cetacean species, releasing individuals from captive or semi-natural environments into suitable original habitats—paired with PRM to evaluate survival—is vital. This approach enhances conservation knowledge and provides demographic and genetic support to wild populations [14]. To date, there have been few significant successes in establishing *ex situ* populations among cetaceans as a strategy to address deteriorating habitat conditions.

The Yangtze finless porpoise (YFP) (*Neophocaena asiaeorientalis asiaeorientalis*) has emerged as a rare success in *ex situ* conservation. It is currently the only cetacean subspecies known to have successfully established self-sustaining *ex situ* breeding population [8]. Since the 1970s, this freshwater population has experienced a rapid population decline due to multiple drivers, including mortality from entanglement in fishing gear, prey depletion and habitat degradation [15]. Despite being subjected to frequent human disturbances [16], the Yangtze River is currently undergoing unprecedented conservation efforts. The implementation of the ‘Ten-year Fishing Ban’ has curbed overfishing and the use of fishing gear, which serve as important drivers of the species’ decline [17]. Previous studies demonstrated that YFPs from the *ex situ* populations can adapt to a more natural environment after undergoing acclimatization training [18]. Here, we report on the release of two acclimatized YFPs into the Yangtze River, along with intensive PRM. This achievement represents a critical milestone in *ex situ* conservation for this population and may provide a model for conserving other endangered riverine species highlighted by the IUCN [19].

## 2. Methods

### (a) Animals and release site

**Figure 1.**
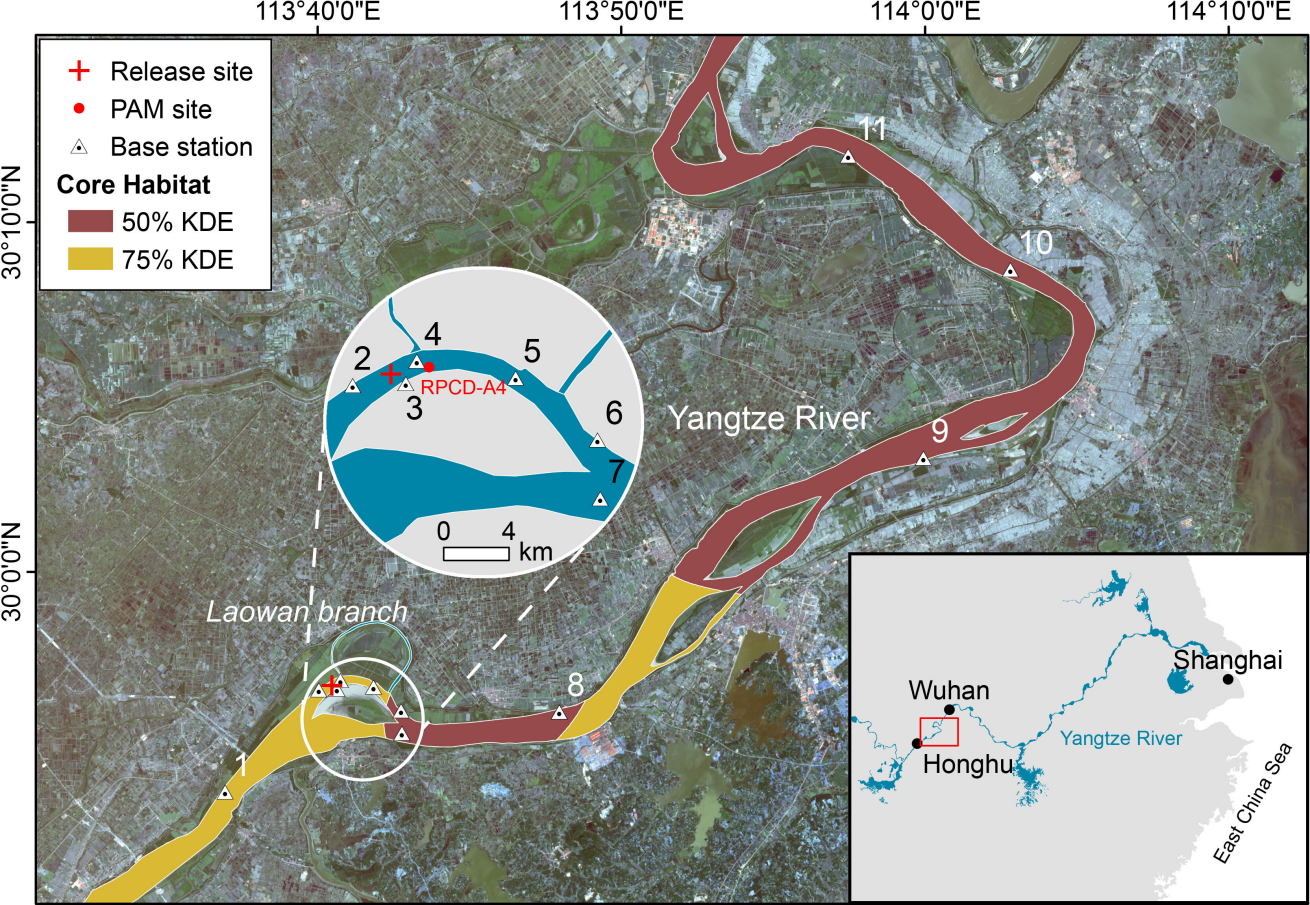
Map of post-release monitoring. Brown and yellow areas represent 50% and 75% core habitats, respectively (see electronic supplementary material). The released Yangtze finless porpoises were subjected to pre-release acclimation in Laowan Branch. Release site is marked with a red cross. A total of 11 base stations, along with a real-time porpoise click detector (RPCD-A4) were deployed in nearby waters. Satellite imagery (photo taken in December 2020) is sourced from Landsat 8−9 OLI/TIRS.

Two male YFPs (T21M42: 135.0 cm, 42.4 kg; T21M02: 127.0 cm, 33.2 kg), originating from the *ex situ* population in Tian-e-Zhou, were captured on 28 April 2021. Following health examinations and the implantation of identification chips, they were transferred to the Laowan Branch for 2 years of acclimatization period. Specifically, these two YFPs freely ranged and foraged within this approximately 7 km long narrow waterway connected to the Yangtze River. Through long-term passive acoustic monitoring (PAM) and behavioural observations, we evaluated whether they possessed foraging capabilities in low fish-density environments and locomotor adaptability to varying water flow velocities comparable to wild individuals. Additionally, we intentionally introduced boat noise disturbances to simulate the human-dominated environment of the Yangtze River, evaluating their behavioural responses to determine adaptation capacity. The results demonstrated successful acclimatization to this Yangtze River-like environment across all measured parameters. [18]. On 25 April 2023, both porpoises were released into the upstream waters of the Middle Branch, near the Laowan Branch (figure 1). This release site was chosen due to its relatively low levels of human activity and the presence of abundant fish resources. It is not part of the main navigation channel, and during the dry season, it hosts a stable group of over 10 local YFPs.

### (b) Tracking with wearable radio tags

**Figure 2.**
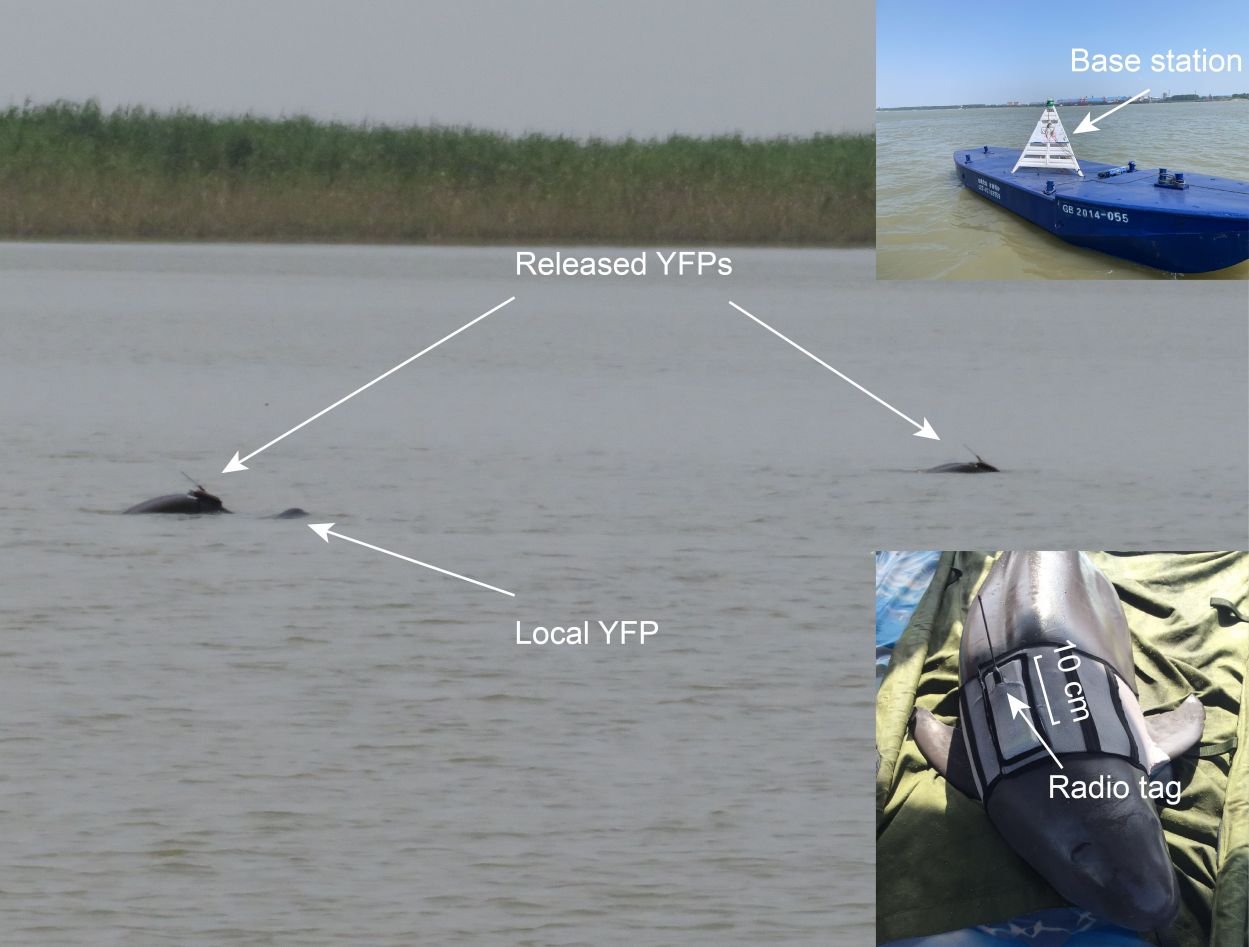
Two released YFPs were photographed with a local individual on the third day post-release. Close-ups show the base station and one YFP (T21M02) wearing a radio tag.

The released porpoises were tracked using a wearable radio tag system. This system comprises a miniature radio tag, affixed to a vest, that transmits signals collected by base stations positioned around the release area (figure 2). The vest is designed to fit the porpoise’s body, securing the tag on the dorsal side to ensure proper attachment for an extended duration. The vest will automatically detach due to material degradation after a period of time to minimize any long-term impact on the animal. When the porpoises surface to breathe, the tags exposed to the air would emit electromagnetic waves, which are collected by the base stations, allowing us to obtain spatiotemporal information on the tagged porpoises. During tracking, the radio tag was programmed to emit signals every 2 s for a 2 min period at 5 min intervals to increase battery life and reduce missed breathing of the animals.

### (c) Stationary passive acoustic monitoring and vessel-based visual surveys

Intensive acoustic monitoring was conducted from 25 April to 2 June 2023, using stationary real-time porpoise click detectors (RPCD-A4, Wuhan Pindu Technology Co., China) equipped with a four channel hydrophone array (figure 1). Visual surveys based on boats were also carried out from 26 to 30 April and 7 to 8 May 2023 (see electronic supplementary material, Methods).

### (d) Yangtze Cetacean Protection Network collection of dead porpoises

The Yangtze Cetacean Protection Network (YCPN) managed by the Institute of Hydrobiology, Chinese Academy of Sciences (IHB, CAS), operates with the support of local governments along the Yangtze River. Local residents report any sightings of dead YFPs to the network. The carcasses are collected and scanned to verify whether the identification chips match the released individuals.

## 3. Results

On 28 April, the two released YFPs were observed interacting with a local porpoise (figure 2). Concurrently, the nearby RPCD-A4 detected the presence of three porpoises, and the radio base stations recorded signals from both released individuals. RPCD-A4 registered 393 YFP echolocation clicks over 9 days, corresponding to 33 distinct porpoise occurrence events (electronic supplementary material, table S2). The estimated group sizes during these events ranged from one to four individuals.

**Figure 3.**
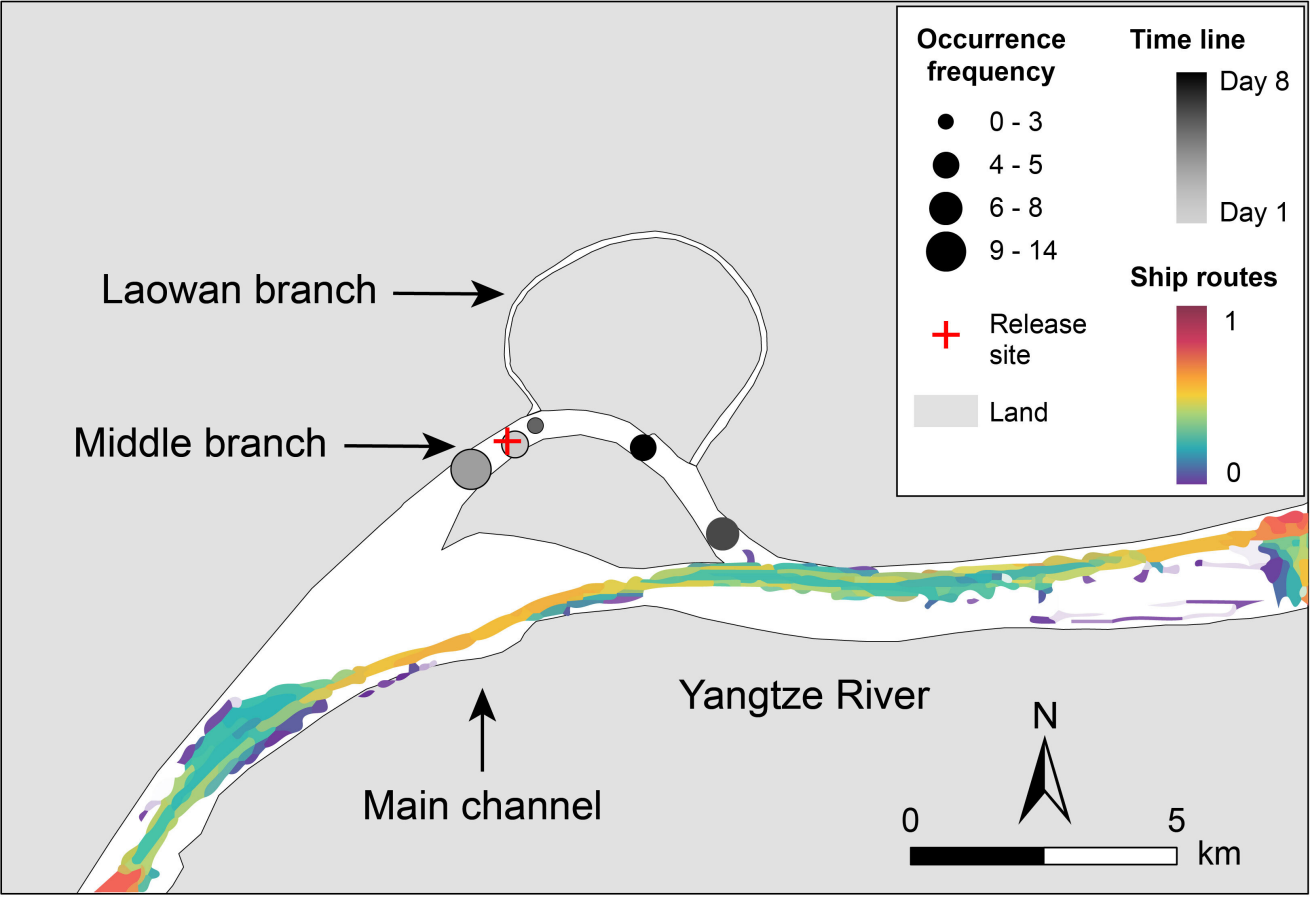
The signals from the released YFPs detected by the base stations. The size of the circles indicates the frequency of signals received by the base stations in the respective areas, while the greyscale colour represents the timeline of signal reception. The colour gradient in the main channel depicts the normalized mean shipping route density for 2022. The shipping data are sourced from marinetraffic.com.

Between 25 April 2023, at 10.21, and 3 May 2023, at 22.57, the base stations received a total of 35 signals from the tags. However, no signals were detected on 27 April or between 30 April and 2 May (figure 3, electronic supplementary material, table S1). On all other days, both porpoises were detected, except on 29 April, when only T21M02’s signals were recorded.

During 5 days of vessel-based visual surveys, a total of 35 individual porpoises were observed across 14 sightings (electronic supplementary material, figure S1). Of these, 92.86% (13 out of 14 sightings) occurred within the 50% core distribution area identified in historical data (see electronic supplementary material).

From 25 April 2023 to 1 March 2025, the YCPN has continuously monitored the river and collected reports of dead YFPs. No identification chips from the two released individuals (T21M42 and T21M02) were found in any of the detectable carcasses collected along the Yangtze River, Dongting Lake or Poyang Lake.

## 4. Discussion

The intensive PRM results indicate that the two YFPs have successfully integrated into the local population in the Yangtze River, occupying suitable habitats. Moreover, no mortality was reported for the released individuals, suggesting the success of this release into the wild. This achievement represents a significant advancement in the conservation of YFPs, which in time, once the habitat is restored and anthropogenic threats have been controlled, will contribute to population recovery.

A critical precondition for successful population reinforcement or reintroduction is the control or mitigation of initial threats in the species’ native habitat [3]. For YFPs, major *in situ* threats include overfishing, fishing gear entanglement and shipping traffic [17]. While it is impractical to substantially reduce shipping traffic on the Yangtze River, known as the ‘Golden Waterway’, the other two risk factors have been effectively controlled following the implementation of the ‘Ten-Year Fishing Ban’ policy. This large-scale, coordinated effort has led to measurable ecological improvements, including the recovery of fish populations [20] and enhanced habitat conditions for YFPs [21]. These positive changes, achieved through strong collaboration among multiple stakeholders, have created a more favourable environment for translocated individuals, significantly increasing their chances of survival and integration into the wild population. Pre-release acclimatization training also confirmed that the two released individuals adapted well to the Laowan Branch, a more natural environment similar to the Yangtze River mainstream [18], which likely contributed to their post-release success.

Cases of PRM for cetaceans are relatively rare and often involve invasive tracking techniques that cause physical damage [9–11]. For larger cetaceans, the physiological damage of invasive tags might be limited, though there have been reports of killer whale (*Orcinus orca*) dying from infections related to invasive devices (https://www.orcaconservancy.org/blog/necropsy-l95-final-report-ahc-case-16-1760). In contrast, small cetaceans like the YFPs, which lack dorsal fins, present additional challenges for secure attachment of invasive devices. Suction-cup tags, used in previous short-term studies [22], often fail to remain attached long enough. To address these challenges, we employed a vest-based wearable radio tag system that minimizes harm and allows for relatively longer-term tracking. In 2018, this system was successfully used to track two YFPs in Poyang Lake for over two weeks (unpublished data), demonstrating the safety and efficacy of this tagging approach.

Our data showed that, by the third day post-release, the two released YFPs had formed a small group with a local individual (figure 2). Interaction with local individuals is known to improve the survival of the translocated animals. Records from the Baiji Aquarium in Wuhan indicate that YFPs rescued from the wild tend to display low appetite and activity levels when first placed in captivity. However, when housed with other captive porpoises, they engage in frequent interactions and quickly regain normal behaviour. Similar patterns have been observed in other species; for instance, interactions between translocated and resident individuals may be one of the factors contributing to the successful reintroduction of bottlenose dolphin (*Tursiops truncatus*) [13]. Conversely, the absence of social interaction may hinder translocation efforts, as seen in the case of Keiko the orca, who ultimately failed to return to the wild, possibly due to the inability to reintegrate into wild pods after release [12].

During their adaptation to the wild environment, the released YFPs exhibited stage-specific distribution patterns. Initially, they stayed near the release site, but gradually expanded their range. This differs from cases involving the release of other cetaceans captured from the wild, as studies have shown that these animals often rapidly disperse from the release site [9–11,23], with some individuals even returning to their original capture areas [13]. The initial site fidelity of the YFPs may be attributed to the impacts of shipping traffic and noise in the main channel (figure 3). After at least 9 days of adaptation (post-May 4, electronic supplementary material, table S2), the porpoises left the vicinity of the release site.

The final radio signal, received on 3 May, likely represents the detachment of the wearable radio tag. After this detachment, visual surveys and PAM continued to detect large groups of porpoises, whose distributions were consistent with visual observation records from 2006 to 2023 in protected areas [24] (see also electronic supplementary material). The YFPs appear to establish their home ranges faster than other animals. A previous study [18] has shown that these two individuals adapted to the Laowan Branch during their pre-release acclimatization training in approximately two months. It is reasonable to infer that early interactions with local individuals may facilitate their smoother integration into the local population and appropriate habitat.

Additionally, we conducted intensive monitoring along the release section’s shorelines through the YCPN. Managed by the IHB and established in 2008, this network spans the Yangtze River basin, including Dongting and Poyang Lakes. The YCPN, characterized by its sustainability, low cost and extensive coverage, leverages government, volunteer and citizen science efforts. From 2007 to 2021, the YCPN has collected 259 stranded and deceased YFPs [25]. The absence of identification chips from the two released YFPs in all collections since their release suggests no accidental mortality has occurred.

Although the PRM was largely successful, several limitations should be noted. Firstly, the wearable radio-tag base station system provided relatively little data, likely due to reduced signal transmission to extend battery life. Moreover, the base stations lacked precise location tracking capabilities. Future PRM should incorporate more accurate tracking methods to determine the activity patterns of released YFPs. Secondly, while the YCPN provides valuable long-term data on the survival of released YFPs, this network is unique to the Yangtze River and may not be applicable to broader marine environments.

The primary goal of some *ex situ* conservation is to eliminate or mitigate risk factors to allow individuals to be released back into their native habitat, rather than establishing a new population elsewhere. The successful release of the YFP represents the world’s first case of *ex situ* cetacean individuals being returned to wild populations. The 2022 YFP population survey has revealed a trend of population recovery [26], reflecting the effectiveness of *in situ* conservation efforts. Meanwhile, the programme may provide additional support for the restoration of the wild population, although the number of individuals released so far remains limited. Furthermore, before general guidelines for the release of *ex situ* individuals can be established, more proactive attempts are necessary. In the future, continued improvements in PRM techniques are essential to achieve long-term and more precise monitoring of released individuals.

## Data Availability

All data presented in this manuscript are available from the Dryad Digital Repository [27]. Supplementary material is available online [28].

## References

[1] Smith D *et al*. 2023 Extinct in the wild: the precarious state of Earth’s most threatened group of species. Science **379**, eadd2889. (10.1126/science.add2889) 36821678

[2] IUCN/SSC. 2014 Guidelines on the use of ex situ management for species conservation. Version 2.0 ed. Gland, Switzerland: IUCN Species Survival Commission.

[3] IUCN/SSC. 2013 Guidelines for reintroductions and other conservation translocations. Version 1.0 ed. Gland, Switzerland: IUCN Species Survival Commission.

[4] Cardillo M, Mace GM, Jones KE, Bielby J, Bininda-Emonds ORP, Sechrest W, Orme CDL, Purvis A. 2005 Multiple causes of high extinction risk in large mammal species. Science **309**, 1239–1241. (10.1126/science.1116030) 16037416

[5] Braulik GT *et al*. 2023 Red‐list status and extinction risk of the world’s whales, dolphins, and porpoises. Conserv. Biol. **37**, e14090. (10.1111/cobi.14090) 37246556

[6] Cañadas A, Vázquez JA. 2014 Conserving Cuvier’s beaked whales in the Alboran Sea (SW Mediterranean): identification of high density areas to be avoided by intense man-made sound. Biol. Conserv. **178**, 155–162. (10.1016/j.biocon.2014.07.018)

[7] Embling CB, Gillibrand PA, Gordon J, Shrimpton J, Stevick PT, Hammond PS. 2010 Using habitat models to identify suitable sites for marine protected areas for harbour porpoises (Phocoena phocoena). Biol. Conserv. **143**, 267–279. (10.1016/j.biocon.2009.09.005)

[8] Mei Z *et al*. 2014 The Yangtze finless porpoise: on an accelerating path to extinction? Biol. Conserv. **172**, 117–123. (10.1016/j.biocon.2014.02.033)

[9] Tyson Moore RB, Douglas DC, Nollens HH, Croft L, Wells RS. 2020 Post-release monitoring of a stranded and rehabilitated short-finned pilot whale (Globicephala macrorhynchus) reveals current-assisted travel. Aquat. Mamm. **46**, 200–214. (10.1578/am.46.2.2020.200)

[10] Dunn C, Claridge D, Herzing D, Volker C, Melillo-Sweeting K, Wells RS, Turner T, O’Sullivan K. 2020 Satellite-linked telemetry study of a rehabilitated and released Atlantic spotted dolphin in the Bahamas provides insights into broader ranging patterns and conservation needs. Aquat. Mamm. **46**, 633–639. (10.1578/am.46.6.2020.633)

[11] Gales R, Alderman R, Thalmann S, Carlyon K. 2012 Satellite tracking of long-finned pilot whales (Globicephala melas) following stranding and release in Tasmania, Australia. Wildl. Res. **39**, 520. (10.1071/wr12023)

[12] Simon M, Hanson MB, Murrey L, Tougaard J, Ugarte F. 2009 From captivity to the wild and back: an attempt to release Keiko the killer whale. Mar. Mammal Sci. **25**, 693–705. (10.1111/j.1748-7692.2009.00287.x)

[13] Wells RS, Bassos‐Hull K, Norris KS. 2006 Experimental return to the wild of two bottlenose dolphins. Mar. Mammal Sci. **14**, 51–71. (10.1111/j.1748-7692.1998.tb00690.x)

[14] White LC, Moseby KE, Thomson VA, Donnellan SC, Austin JJ. 2018 Long-term genetic consequences of mammal reintroductions into an Australian conservation reserve. Biol. Conserv. **219**, 1–11. (10.1016/j.biocon.2017.12.038)

[15] Huang J *et al*. 2020 Population survey showing hope for population recovery of the critically endangered Yangtze finless porpoise. Biol. Conserv. **241**, 108315. (10.1016/j.biocon.2019.108315)

[16] Mei Z *et al*. 2021 Mitigating the effect of shipping on freshwater cetaceans: the case study of the Yangtze finless porpoise. Biol. Conserv. **257**, 109132. (10.1016/j.biocon.2021.109132)

[17] Wang D. 2009 Population status, threats and conservation of the Yangtze finless porpoise. Chin. Sci. Bull. **54**, 3473–3484. (10.1007/s11434-009-0522-7)

[18] Qiu J *et al*. 2023 The first case of reintroduction and behavioral adaptability of Yangtze finless porpoise. Acta Hydrobiol. Sin. **47**, 1709–1718. (10.7541/2023.2023.0064)

[19] IUCN. 2020 *Ex situ* options for cetacean conservation: report of the 2018 workshop, Nuremberg, Germany. Occasional paper of the iucn species survival commission, (eds BL Taylor *et al*.). Gland, Switzerland: IUCN.

[20] Feng K *et al*. 2023 Direct and indirect effects of a fishing ban on lacustrine fish community do not result in a full recovery. J. Appl. Ecol. **60**, 2210–2222. (10.1111/1365-2664.14491)

[21] Wang ZT, Duan PX, Akamatsu T, Wang KX, Wang D. 2024 Increased Yangtze finless porpoise presence in urban Wuhan waters of the Yangtze River during fishing closures. Ecol. Evol. **14**, e11247. (10.1002/ece3.11247) 38584767 PMC10994980

[22] Kimura S, Akamatsu T, Wang D, Li S, Wang K, Yoda K. 2013 Variation in the production rate of biosonar signals in freshwater porpoises. J. Acoust. Soc. Am. **133**, 3128–3134. (10.1121/1.4796129) 23654415

[23] Pulis EE, Wells RS, Schorr GS, Douglas DC, Samuelson MM, Solangi M. 2018 Movements and dive patterns of pygmy killer whales (Feresa attenuata) released in the Gulf of Mexico following rehabilitation. Aquat. Mamm. **43**, 555–567. (10.1578/am.44.5.2018.555)

[24] Xu Z, Zhang Y, Liu X, Chen M, Mei Z. 2025 Distribution and influence on river morphology of the Yangtze finless porpoise in the Xinluo section of the Yangtze Baiji National Nature Reserve. Acta Hydrobiol. Sin **49**, 052505. (10.7541/2025.2024.0362)

[25] Wang R. 2022 Stranding information analyses and implications for in situ conservation of the Yangtze finless porpoise. MSc thesis, Institute of Hydrobiology, Chinese Academy of Sciences, Wuhan, China.

[26] Hao Y, Tang B, Mei Z, Zheng J, Wang K, Wang D. 2024 Further suggestions on conservation of the Yangtze finless porpoise based on retrospective analysis of the current progress. Acta Hydrobiol. Sin. **48**, 1065–1072. (10.7541/2024.2024.0020)

[27] Qiu J *et al*. 2025 Data from: rebuilding the critically endangered Yangtze finless porpoise population: successful release from an ex situ conservation programme. Dryad. (10.5061/dryad.k0p2ngfj5)40460860

[28] Qiu J, Zheng Y, Fan F, Zheng J, Zeng Q, Xu Z *et al*. 2025 Supplementary material from: Rebuilding the Critically Endangered Yangtze finless porpoise population: successful release from an ex situ conservation programme. Figshare. (10.6084/m9.figshare.c.7809818)40460860

